# Open drug discovery for the Zika virus

**DOI:** 10.12688/f1000research.8013.1

**Published:** 2016-02-09

**Authors:** Sean Ekins, Daniel Mietchen, Megan Coffee, Thomas P Stratton, Joel S Freundlich, Lucio Freitas-Junior, Eugene Muratov, Jair Siqueira-Neto, Antony J Williams, Carolina Andrade

**Affiliations:** 1 Collaborations in Chemistry Inc, Fuquay-Varina, NC, USA; 2 Collaborations Pharmaceuticals Inc., Fuquay-Varina, NC, USA; 3 Collaborative Drug Discovery Inc., Burlingame, CA, USA; 4 Open Knowledge Foundation Deutschland e.V., Berlin, Germany; 5 The International Rescue Committee , NY, NY, USA; 6 Department of Pharmacology, Physiology and Neuroscience, Rutgers University-New Jersey Medical School, Newark, NJ, USA; 7 Division of Infectious Diseases, Department of Medicine, and the Ruy V. Lourenço Center for the Study of Emerging and Re-emerging Pathogens, Rutgers University–New Jersey Medical School, Newark, NJ, USA; 8 Chemical Biology and Screening Platform, Brazilian Laboratory of Biosciences (LNBio), CNPEM, Campinas, Brazil; 9 Laboratory for Molecular Modeling, Division of Chemical Biology and Medicinal Chemistry, Eshelman School of Pharmacy, University of North Carolina, Chapel Hill, NC, USA; 10 Skaggs School of Pharmacy and Pharmaceutical Sciences, University of California San Diego, San Diego, CA, USA; 11 ChemConnector, Wake Forest, NC, USA; 12 LabMol - Laboratory for Molecular Modeling and Drug Design, Faculty of Pharmacy, Federal University of Goias, Goiânia, Brazil

**Keywords:** Zika, microcephaly, drug discovery, flavivirus, Aedes, dengue, yellow fever, ebola

## Abstract

The Zika virus (ZIKV) outbreak in the Americas has caused global concern that we may be on the brink of a healthcare crisis. The lack of research on ZIKV in the over 60 years that we have known about it has left us with little in the way of starting points for drug discovery. Our response can build on previous efforts with virus outbreaks and lean heavily on work done on other flaviviruses such as dengue virus. We provide some suggestions of what might be possible and propose an open drug discovery effort that mobilizes global science efforts and provides leadership, which thus far has been lacking. We also provide a listing of potential resources and molecules that could be prioritized for testing as *in vitro* assays for ZIKV are developed. We propose also that in order to incentivize drug discovery, a neglected disease priority review voucher should be available to those who successfully develop an FDA approved treatment. Learning from the response to the ZIKV, the approaches to drug discovery used and the success and failures will be critical for future infectious disease outbreaks.

## Background - Zika virus epitomizes a neglected disease

We did not have to wait too long for the next virus to make global news ^1^. A fast follower to the Ebola virus (EBOV) epidemic that killed over 11,000 in Africa ^2– 4^ during 2014–2015, the Zika virus (ZIKV) has been suggested to have pandemic potential ^5– 7^. While EBOV was likely not a new name to most, ZIKV only became part of most people’s vocabulary in the past few weeks in the Western world, indicating a lack of knowledge of the virus. The ZIKV is an arthropod-borne flavivirus of the family *Flaviviridae*, phylogenetically close to dengue virus and yellow fever, transmitted by *Aedes* mosquitoes ^8^, that usually causes a mild dengue-like illness with possibly fever, joint pains, rash, and/or swollen lymph nodes ^9^ and has been more recently associated with *rare* Guillain-Barré syndrome ^10^. It was neglected because there are multitudes of viruses and ZIKV had not seemed to cause severe pathology as it is thought to be the case now. However, the dramatic increase in the number of cases of babies born with microcephaly, especially in Brazil ^11^ and possibly associated with this virus, brought the ZIKV to the immediate attention of the West as it has spread. Cases of microcephaly had never been seen as a risk. It is possible that many children did not make it to adulthood if they were infected with ZIKV. It may have been that common. So few women would be infected during pregnancy. Those who were would be few in number and sporadically, not all at once, so the risk, if present, would have been hard to identify. Brazil without immunity in the population saw large numbers infected immediately as the virus was amplified in the population, resulting in thousands of pregnant women infected at once. Initially the Brazilian Ministry of Health advised reporting diagnosed cases of Zika as dengue, since the symptoms were in most of the cases similar to a mild case of the latter. After the association with microcephaly was announced, they revised the advice and recommended reporting the diseases independently, meaning that the official initial numbers of ZIKV incidence are most likely underestimated. The first baby in the USA born with ZIKV occurred on January 16 ^th^, 2016 ^12^. Although not yet scientifically proven ^13^, the relationship between ZIKV infection during pregnancy and microcephaly is strongly suspected ^14^. Unlike EBOV, the ZIKV has travelled around the globe and has affected many countries ^9^. It has also resulted in the World Health Organisation (WHO) moving much faster than they did against EBOV ^15^, although it is unclear whether they are willing to take a leadership role ^16, 17^. Which begs the question who is going to manage the global response to the virus? The WHO Director-General declared on February 1 ^st^, 2016 that the cluster of microcephaly cases and other neurological disorders reported in Brazil constitutes a Public Health Emergency of International Concern ^14^ (PHEIC), it has therefore been identified as a problem for the entire world to deal with.

What is clear is that the ZIKV has been relatively ignored by researchers for over 60 years, with just 150 articles in PubMed at the time of writing since the original description of the virus was published in 1952 ^18^, although it was originally isolated in 1947 in Uganda, Africa ^8^. While there are some sources of information on ZIKV such as protein sequences etc. ( Table 1), a look in the Protein Data Bank (PDB), ChEMBL and PubChem databases is more despairing at the time of writing, with zero crystal structures for proteins from this virus or any assays that deal with targets or in whole cells. This translates to no molecules that have been screened against ZIKV targets and certainly no approved drugs that have been tested either *in vitro* or *in vivo* in relevant animal models which are also absent. Analysis of patents also suggests there are no specific molecules identified as active against ZIKV, although there are several patents on compounds for dengue ^19^. Based on these observations, ZIKV should clearly be labeled as a “neglected disease”.

**Table 1.** A list of data sources and repositories for Zika virus information.

Source name	Website
Wikidata	71
University of Minnesota Center for Disease Research and Policy	72
Centers for Disease Control	73
Figshare	74
PLOS Collections – Zika virus	75
F1000Research Zika and arbovirus outbreaks channel	76
Peptidase database	77
Institut Pasteur	78
World Health Organization	79

According to NIH RePORTER ^20^, there have been no projects funded to date to specifically address ZIKV. The NIH NIAID has responded by suggesting they would consider submissions for grants that address this virus ^21^. There has been an acceleration in the opportunities for research, especially after the declaration of ZIKV as a PHEIC, but given the inherent need for ethical review and design of trials, this process cannot move as quickly as many wish. For example it is unlikely that any NIH funded projects would start until much later in the year, which would delay potential discoveries. So far, the Bill and Melinda Gates Foundation and the Wellcome Trust have not announced any funding for ZIKV research. If we are to address preventing further spread of this virus, we have to move much faster and be coordinated in our response, maybe implementing a disruptive approach to moving towards a potential cure. It appears the NIH is not actively working on their own *in vitro* assay for ZIKV. Those extramural scientists already funded to work on similar viruses may be in a position to shift resources or perhaps may have access to alternative funding sources that could be utilized. This does beg the question how we can achieve faster dispersal of funding resources that can be mobilized in public health emergencies like this, whether through the WHO or World Bank or others. Dedicated teams of experts that can tackle such challenges perhaps also need to be convened to see if they can help lead global efforts. These need to be initiated in days and not weeks or months. Clearly, this did not happen with EBOV, and so far, it has not happened with ZIKV, at least in the USA.

## Kickstarting ZIKV drug discovery

We, and others, have already suggested what steps perhaps could be taken to kick start a drug discovery program in such a circumstance as this ^22^. There is significantly more information on the related flavivirus, dengue virus, with several high throughput screens and computational drug discovery efforts that have resulted in small molecule hits ( Table 2). If we want a molecule to reach the clinic quickly for ZIKV, probably the most expedient method may be to repurpose FDA and/or EU approved drugs ^23^ ( Table 2). This has been an approach that has led to new *in vitro* or *in vivo* active compounds of clinical relevance in a number of cases ^24, 25^. The challenge, of course, is how to do this when there is zero prior work either *in vitro* or *in silico*. We could certainly leverage the data and models (including computational models) that are available for dengue virus as a starting point, but ideally we need to generate some data for compounds screened against the ZIKV. It is *possible* that there are already data available sitting behind academic or corporate firewalls, and ideally these need to be released to the world for examination (preferably as open data). We now offer some steps which could be taken immediately:

**Table 2.** List of potential compounds to test.

Compound source	Compounds
FDA approved antiviral drugs	29
FDA drugs that are not antivirals but have shown antiviral activity	Antimalarials versus Ebola; Quinacrine, Pyronaridine ^24^ Chloroquine and Amodiaquine ^51^ Kinase inhibitors ^32, 80^ Chlorcyclizine ^81^ NTCP inhibitors vs HepB ^82– 86^
FDA approved drugs active *in vitro* or *in vivo* vs dengue virus.	Quinacrine, Berberine ^87^ Amodiaquine ^88^ Prochlorperazine ^89^
Other compounds from HTS screens vs dengue virus, yellow fever etc.	H-89, MPP, BIBU 1361 ^87^ Diverse molecules ^39– 45^
Compounds from ChEMBL datasets	90– 95
Compounds from PubChem	96– 98

A first step would be to develop a whole cell or target-based ZIKV *in vitro* assay that would be amenable to medium to high throughput screening. This would need to be undertaken in BSL 2 facilities, which may limit the number of laboratories that can perform the screening, though expedited data sharing could allow others to help with analyses, informatics, contextualization, quality control and related aspects of the work and thereby accelerate it ^26^. In particular, high content screening can considerably speed the discovery and development of new drugs for Zika chemotherapy. By precluding the need for validated targets, cell-based screening enables the discovery of compounds that can inhibit virus entry and/or replication in human cells, by either deploying fluorescent protein-tagged ZIKV or using antibodies as probes for detection of viral proteins expressed in host cells. This approach has been successfully applied to flaviviruses such as Hepatitis C and dengue ^27^. A simpler assay that could be easily implemented in laboratories with isolated virus not requiring expensive automation or instrumentation would be a viability assay for host cells infected with the virus. Viability markers such as resazurin (Sigma R7017) would be an inexpensive viability marker that could be assessed by colorimetric or fluorimetric readout after being converted to resofurin by the mitochondria of the host cell. The assay design could start with plating host cells such as Vero in a micro-well plate and adding the test compounds followed by addition of ZIKV. Adding the compounds before the virus allows for the detection of invasion inhibitors. Incubation for 4 to 5 days would be enough to eliminate all the cells in the wells untreated or with ineffective compounds. Resazurin solution would be added on the last day of the assay and incubated for at least 1 hour. Effective antiviral compounds would prevent cell death and could be detected by the reduction of resazurin to resofurin by the change in color (from purple to pink) or by fluorescence readout with excitation at 515nm. This assay would not require any genetic manipulation of the virus, and could be implemented with different clinical isolates, being also amenable to mid-high throughput scale screening.

**Figure 1.**
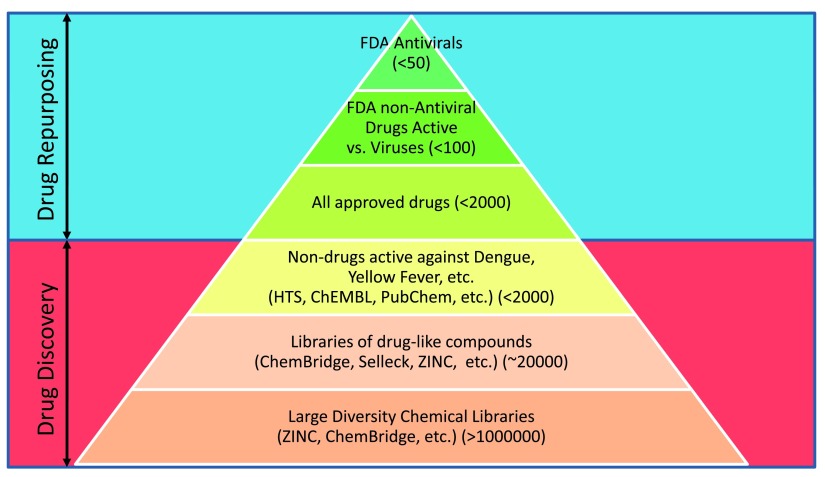
Compounds and chemical libraries suggested for testing against Zika virus.

A second step that seems appropriate would be to test drugs and other chemical compounds in the assay developed in step 1. We have summarized all the compounds and chemical libraries suggested for testing against ZIKV in Figure 1. We also sorted them by the priority level for testing. The number of chemicals at each level is given in parenthesis. Here we want to emphasize that we strongly support the idea of drug repurposing in general because it is the quickest way to the introduction of a drug into the market and its use in patients ^23, 28^. Due to the absence of any relevant treatment, this is especially important for the rapid discovery of a drug against ZIKV. We also suggest to start from the 48 FDA-approved antivirals ( Table 2) ^29, 30^. Special priority should be given to the antivirals that were shown to be active against other flaviviruses such as dengue virus ( Supplementary material S1), yellow fever, Japanese encephalitis, etc., and to a lesser degree, against other members of the *flaviviridae* family like Hepatitis C ( Table 2). In addition to antivirals, we could also recommend approved non-antiviral drugs that have shown antiviral activity. Moreover, being inspired by the discovery of anti-influenza properties of brinzolamide ^31^ and activity of toremifene against Middle East respiratory syndrome coronavirus infection ^32^, and EBOV ^33^ we also recommend to test, in addition to antiviral compounds, all other marketed drugs. This will increase our chances to find a treatment against ZIKV and will preserve all the benefits of drug repurposing. The main reason preventing us from this approach could be potential low throughput of the developed assay. Another obstacle is the cost of these drugs or corresponding drug libraries, e.g., Prestwick Chemical Library ^34^. However, this could be overcome by in-kind donation of drug samples from big pharmaceutical companies (Pfizer, GSK, etc.) or chemical manufacturers (Prestwick, ChemBridge, Selleck, etc.). Other compounds that are not approved drugs could be also tested ( Figure 1). We believe it is still better to start from compounds already approved or undergoing clinical trials (e.g. NIH clinical collection) but not yet approved by FDA and chemicals active against dengue, yellow fever, etc. The latter could be found in HTS assays, ChEMBL, PubChem, etc., and are summarized in Table 3. Perhaps less attractive but still a reasonable step is the use of focused libraries of drug-like compounds and, as a last resort, large diverse chemical libraries containing millions of compounds.

**Table 3.** Targets in Zika virus with homology to dengue virus.

Target	References
Envelope glycoprotein	45
Proteases NS2B3 and NS3	99– 101
NS3 helicase	102, 103
NS5 methyltransferase (e.g. Guanylyltransferase)	43, 102
NS5 RNA-dependent RNA polymerase	102, 104, 105
Host factors	106– 108

A third approach would be to explore the complete genome of ZIKV ^35, 36^ or the recently published genome for ZIKV circulating in the Americas ^37^ to apply a target-based chemogenomics approach ^38^ in order to identify approved drugs that may be active against the ZIKV for testing *in vitro*.

**Figure 2.**
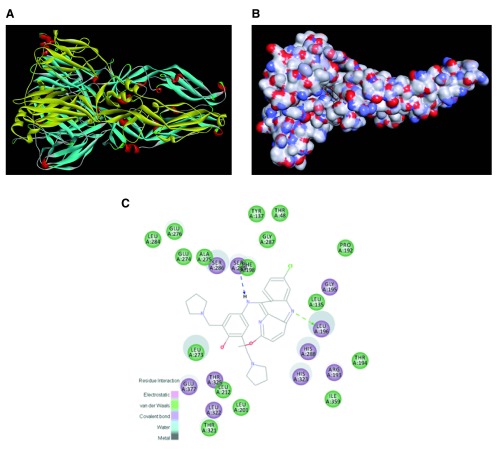
Homology model created for the Zika virus envelope protein. **A**. Complete protein shown as a ribbon diagram (generated in Discovery Studio). **B**. Pyronaridine shown docked into the subunit A homology model, small molecule colored by atom, protein colored by atom charge. **C**. 2D interaction plot for pyronaridine.

A fourth approach would be to develop homology models for ZIKV proteins that are similar to those targeted by molecules that are active against the dengue virus ^39– 45^ ( Table 3). This would enable structure-based approaches such as docking to significantly narrow down the number of compounds for eventual testing. As this is one of the most accessible approaches at the present time, as an example, we have used freely available online software to create a homology model of the ZIKV envelope protein ( Supplementary material S2) that can be used to dock compounds and score them in order to prioritize compounds for *in vitro* testing ( Figure 2; Supplementary material S3– Supplementary material S6). Alternatively, we could turn to libraries of commercially available, drug-like small molecules for screening *in silico* and then *in vitro*. An expedited path to their optimization toward pre-clinical candidates could rely on publicly available computational machine learning models for critical physiochemical and ADME properties that we and others have made available to the scientific community ^46, 47^.

A fifth step would be to understand the mechanism and target of any compounds derived from whole cell screening and confirm compounds that have on-target activity when identified by target-based *in vitro* or *in silico* approaches. This might be enabled by using similarity of molecules that have been identified to have activity against targets in other species using target prediction software ^48, 49^. Potentially promising molecules could also be screened against other viruses (flaviviruses or others) to identify whether they can be used across a whole virus class or have even broader antiviral activity.

A final step would be to test compounds in an animal model of ZIKV infection such as the mouse ^50^ initially. It is unclear whether larger animal models have been developed and tested yet. Once a suitable model has been validated, we stress the importance of assaying a significant number of promising early candidates (if they exist) and also examining opportunities for drug repurposing. Despite the significant advances in the biological sciences over the last 70 years, we must not forget that many of our current anti-infectives arose during the World War II drug discovery effort. At that point in history, small molecules were synthesized and tested *in vivo* with little delay. Certainly while we cannot ignore requisite animal toxicity studies and guidelines when devoting animals to such studies, we must also not ignore the goal post: *in vivo* demonstration of efficacy.

**Figure 3.**
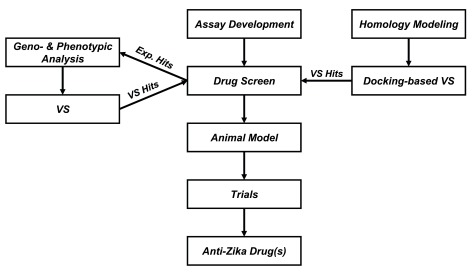
Proposed workflow for rapid drug discovery against Zika virus. Exp = experimental; VS = virtual screen.

The overall proposed workflow for rapid drug discovery against ZIKV is represented in Figure 3. We suggest to start from screening *in vitro* assay (preferably medium- or high-throughput) development with subsequent testing of approved antivirals or other drugs. If drug repurposing will not work, other compounds could be prioritized for testing by docking-based virtual screening using developed homology model or by geno- and phenotypic analyses. It is also possible that this could also be done in parallel using compounds derived from docking prioritized for testing *in vitro*. Subsequent steps are traditional for any drug discovery pipeline and include development of animal models, clinical trial, and in case of success, manufacturing, marketing, and distribution of a drug against ZIKV.

Despite years of knowing of the dangers of Ebola, research into low-hanging fruit - drugs that are FDA approved and perhaps already even indicated in the care of Ebola patients - was limited. Promising leads among FDA approved drugs were identified by Peter Madrid *et al.* using *in vitro* cell culture assays with Ebola ^51^. In light of this paper, Médecins Sans Frontières (MSF) apparently looked at amodiaquine, as it was listed by the WHO as a potential drug, and was already used (as artesunate-amodiaquine) empirically in about half of Ebola patients to treat any malaria infections in suspected or confirmed patients (including almost all patients in Sierra Leone, where it was on the country's protocol). The other patients generally were treated with another malaria medication, artesunate-lumefantrine, which had not been found to have potential activity against Ebola. Work by members of our group showed that a pharmacophore potentially could describe the inhibition of EBOV by amodiaquine and three other compounds identified from published screens ^52^. However, this promising lead in the form of amodiaquine identified before the outbreak was not followed up in initial drug therapy against Ebola. New, unapproved drugs and other therapies, like blood plasma, were prioritized with rather disappointing results ^53– 56^. Later, MSF looking retrospectively showed that during a brief period where stocks of artesunate-lumefantrine were depleted in Liberia and were replaced with artesunate-amodiaquine, mortality dropped by 31% ^57^. Interestingly, dengue also has had potential drugs identified among already approved FDA drugs *in vitro*, including amodiaquine and quinacrine ( Table 2).

We should perhaps also analyze whether vaccines for other viruses (e.g. dengue) may be useful against Zika, although in the past the yellow fever vaccine was not ^58^. Questions to address include whether those previously exposed to dengue virus have an increased propensity to ZIKV.

In addition to development of a drug for ZIKV, complementary parallel efforts should be undertaken in order to understand the virus structure, function, and especially its burden on the human health, including the mechanisms of its pathogenesis and neurological abnormalities. The scientific community needs further information to clarify the dangers created by ZIKV: (i) is ZIKV the only or the main reason for the rise in microcephaly cases and Guillain-Barre syndrome?; (ii) is ZIKV dangerous only to pregnant women and what is the probability to have the above mentioned disorders while being infected?; (iii) what is the mutation potential of the virus?; etc. We need to make sure we are addressing the most important aspects of the disease and not tilting at windmills.

## ZIKV: An opportunity for collaboration

The ZIKV represents an example where national and international readiness needs to be addressed. Clearly, this is also an opportunity to demonstrate the impact of scientific collaboration that could benefit from the mediation of open scientific exchange via open data, open interpretation and step-by-step iterative progress. We propose that open access journals set up Zika repositories and that traditional science publishers could also open up their articles on dengue and related flaviviruses as a means to spur more research and make the literature accessible. It will be important to coordinate these scientific responses to avoid repetition and create these open repositories (e.g. Wikidata – Table 1), but as with many crowdsourcing-based approaches, openness itself will be the primary enabler for exchange and progress. While such initiatives in the past have been limited to malaria and tuberculosis ^59– 62^, diseases which kill millions annually, they have not been widely applied to emerging viruses and neglected tropical diseases. There is no time like the present to see whether these efforts can be brought to bear with great immediacy. The health and lives of a large population of newborns and children is at risk. This may also lead to improved approaches for the next global pandemic.

## How do we incentivize ZIKV drug discovery

To get more companies and groups working on Zika drug discovery ^63^, we propose that this neglected disease should qualify for a neglected disease priority review voucher ^64– 67^. While *Filoviridae* are covered, (e.g. Ebola since 2014), the flavivirus Zika is not. It would need US Senate approval to extend to additional viruses. This obviously takes some effort but Ebola was added at the height of the outbreak, so it is not impossible. The value of these vouchers and those for pediatric rare diseases ^68^ is continuing to increase, so they add a meaningful financial incentive that could bring companies into the effort.

## Summary

The neglected disease space has many issues, including shortage of funds, which has driven us to look for opportunities in using computational approaches ^69^ that have been applied to drug repurposing. The recent ZIKV outbreak reasserts the importance of preparedness for new viruses. While we have a whole array of impressive molecular biology technologies at our disposal, our ability to quickly identify and test molecules that might possess antiviral activity is severely hampered by lack of appropriate *in vitro* assays in place for Zika. We are starting from scratch even though we have known of this virus for over 60 years. This will be an important test of our ability to organize and ramp up efforts that should have been triggered by the Ebola outbreak. However, there should be a warning from that experience, as the big pharmaceutical companies played no role, and it was left to biotechs to field their highly experimental approaches. So for all of the merging of pharmaceutical companies in the last decade, we are possibly weaker for it. Clearly, they too are not willing to pursue a costly antiviral approach, unless there is a substantial financial incentive, and the priority review voucher may fit the bill. And for all the US government’s efforts at being prepared with organizations like the Biomedical Advanced Research and Development Authority (BARDA), who have invested billions of dollars in vaccine readiness for influenza and emerging threats, we are clearly not ready yet. We have herein proposed several approaches that could be actionable now with a bare minimum of resources and funding. It may be the case that we already have FDA and EU approved drugs that while showing activity against other viruses may have a role to play in further testing for efficacy against Zika. While we could develop incredibly sophisticated *in vitro* models for the ZIKV, all that may be needed is a simple readout as to whether a compound has antiviral activity or not. It should certainly not be forgotten that single agent therapies can be overcome quickly by drug resistance, and so from the very beginning, we may want to consider combination therapies like those used in HIV ^70^. We can learn from antiviral drug discovery in the past and try not to repeat the same failures again. The health of a future generation may very well depend on the decisions we make now and our willingness to collaborate to find a cure.

## Figures and Tables

**Table:** 

Molecule	LibDockScore
Colistin	180.85
Ritonavir	180.76
Pepstatin A	171.92
Indinavir	170.23
Deferoxamine	168.53
Lanatoside C	165.79
Dihydroergotamine	161.16
Saquinavir	161.02
Nadide	160.40
Avermectin B1a	159.79

**Table:** 

Molecule	LibDockScore
Halofantrine	152.27
Quinidine	111.02
Amodiaquine	108.83
Chloroquine	108.80
Mefloquine	99.01
Primaquine	95.77

## Supplementary material

Supplementary material S1. Structures of known dengue virus inhibitors as sdf.

**Supplementary material S2.** Method and results for Homology model and docking for Zika virus envelope protein.

### Homology model

The following sequence of the Zika virus envelope protein (E) was taken from the polyprotein ^109^, where the part corresponding to E is from residues 291–592, while the IG-like domain III is from residues 601–693 as shown below

														           ircigvsnrd
301 fvegmsggtw vdvvlehggc vtvmaqdkpt vdielvtttv snmaevrsyc yeasisdmas
361 dsrcptqgea yldkqsdtqy vckrtlvdrg wgngcglfgk gslvtcakfa cskkmtgksi
421 qpenleyrim lsvhgsqhsg mivndtghet denrakveit pnspraeatl ggfgslgldc
481 eprtgldfsd lyyltmnnkh wlvhkewfhd iplpwhagad tgtphwnnke alvefkdaha
541 krqtvvvlgs qegavhtala galeaemdga kgrlssghlk crlkmdklrl kgvsyslcta
601 aftftkipae tlhgtvtvev qyagtdgpck vpaqmavdmq tltpvgrlit anpviteste
661 nskmmleldp pfgdsyivig vgekkithhw hrsgsti

Swiss-Model ^110^ was used to construct a homology model using the Dengue crystal structure 4gsx as a template (58.69 identity) ^111– 114^.

The complete homology model of subunit A was then used in Discovery Studio version 4.1 (Biovia, San Diego, CA) and the ‘prepare protein’ protocol was used before the ‘Dock ligands’ protocol. Selected molecules were initially docked using a docking sphere of 13 angstroms. The proposed binding site was centered on residues 270–277 and a site sphere created (coordinates 17.07, -21.94, 25.70) with 13 Å diameter. The protocol included 10 hotspots and docking tolerance (0.25). The FAST conformation method was also used along with steepest descent minimization with CHARMm. Further parameters followed the default settings. Out of 3 compounds of interest initially docked, pyronaridine had the highest LibDockScore score of 142. Quinacrine – (a known FDA approved drug that has shown activity against Dengue (IC _50_ 0.55μM – 87) has a docking score of 128. The well known antiviral Ribavirin has a docking score of 101. These predictions suggest that Quinacrine may be targeting this protein and that other antimalarials may also be worth testing.

The Prestwick Chemical Library ^34^ of 1280 molecules was first filtered to remove salts then this was docked in the protein as described above.

The top 10 docked molecules from the Prestwick Chemical Library ( S3), as identified using the best scored conformation, includes 3 antivirals: ritonavir, indinavir and saquinavir. Selected antimalarials from the Prestwick Chemical Library ( S4) suggest that these molecules may be worth further testing *in vitro* versus Zika virus given their availability (alongside pyronaridine and quinacrine).

**Supplementary material S3.** The top 10 docked molecules from the Prestwick Chemical Library.

**Supplementary material S4.** Selected antimalarials docked in the Prestwick Chemical Library.

Supplementary material S5. PDB file for homology model.

Supplementary material S5. PDB file for homology model.
