# Supplementary material for: Open drug discovery for the Zika virus
Source: F1000Res. 2016 Feb 9;5:150. [Version 1] doi: 10.12688/f1000research.8013.1 (PMC4841202; doi:10.12688/f1000research.8013.1)
Supplement: Peer review history

# Peer review: Referee response for version 1

**DOI:** 10.5256/f1000research.8622.r13489

**Published:** 2016-04-20

**Authors:** Wade Blair

**Affiliations:** 1 Department of Antiviral Research, Merck Research Laboratories, Merck & Co., Inc., West Point, PA, USA

The authors highlight the urgency and advocate for a collaborative approach to Zika virus drug discovery and development. Advocacy for emerging diseases is always a worthy pursuit and the open drug discovery model suggested by the authors to tackle Zika virus warrants further discussion.  

The authors go on to outline possible drug discovery strategies, with an emphasis on repurposing FDA or EU approved drugs as a faster path to approval of Zika virus therapeutics. Although this is an attractive approach in theory and should be pursued, it is more likely that lead molecules would be identified from this effort rather than effective Zika therapeutics ready for approval. In this short article format, it is difficult for the authors to adequately cover the different drug discovery approaches they recommend. For example, identifying targets and mechanisms for inhibitors identified from a whole cell screen can be complicated and the authors do not discuss the key challenges typically encountered with such a screening campaign. In addition, one very important aspect that is missing from the discussion is drug safety, particularly given that pregnant women in Zika endemic areas represent the population with the highest unmet medical need. The authors should address the hurdles associated with developing drugs with the requisite safety profile for this vulnerable population.

I have read this submission. I believe that I have an appropriate level of expertise to confirm that it is of an acceptable scientific standard.

# Peer review: Referee response for version 1

**DOI:** 10.5256/f1000research.8622.r13487

**Published:** 2016-04-20

**Authors:** John Lowe

**Affiliations:** 1 JL3Pharma LLC, Stonington, USA

The article provides a specific set of recommendations for discovering a new small molecule drug to treat Zika virus infection. Figure 1 in the article summarizes this process, beginning with setting up appropriate assays and screening known anti-viral drugs, especially those already characterized against dengue, another member of the family of viruses to which Zika belongs (Figure 3 is a nice summary of the proposed workflow). The authors also suggest ways to incentivize this effort, and organize it as a collaboration.  All these strategies make sense and are well precedented and documented in the article.  Overall, I recommend the article for approval based on its well-founded analysis of a strategy for addressing a major public health concern.

I have read this submission. I believe that I have an appropriate level of expertise to confirm that it is of an acceptable scientific standard.

# Peer review: Referee response for version 1

**DOI:** 10.5256/f1000research.8622.r13198

**Published:** 2016-04-19

**Authors:** Nicholas Meanwell

**Affiliations:** 1 Department of Discovery Chemistry, Bristol-Myers Squibb Research and Development, Wallingford, CT, USA

This article provides a blueprint for a collaborative approach to identifying inhibitors of not only Zika virus but any emerging viral pathogen. Developing a collection of, for example, nucleoside and nucleoside analogue drugs that could be quickly screened would be a useful starting point but developing screens and a detailed understanding of the virus, analogous to that accomplished when the coronavirus SARS emerged, makes considerable sense.

I have read this submission. I believe that I have an appropriate level of expertise to confirm that it is of an acceptable scientific standard.
